# White and other fur colourations and hybridization in golden jackals (*Canis aureus*) in the Carpathian basin

**DOI:** 10.1038/s41598-023-49265-0

**Published:** 2023-12-11

**Authors:** Nóra Ninausz, Péter Fehér, Erika Csányi, Miklós Heltai, László Szabó, Endre Barta, Péter Kemenszky, Gyula Sándor, Ferenc Jánoska, Mihály Horváth, Szilvia Kusza, Krisztián Frank, László Varga, Viktor Stéger

**Affiliations:** 1 Department of Genetics and Genomics, Institute of Genetics and Biotechnology, Hungarian University of Agriculture and Life Sciences, Gödöllő, Hungary; 2 Faculty of Forestry, University of Sopron, Sopron, Hungary; 3 Department of Wildlife Biology and Management, Institute of Wildlife Management and Nature Conservation, Hungarian University of Agriculture and Life Sciences, Gödöllő, Hungary; 4 SEFAG Zrt., Kaposvár, Hungary; 5 Centre for Agricultural Genomics and Biotechnology, Faculty of Agricultural and Food Sciences and Environmental Management, University of Debrecen, Debrecen, Hungary; 6 MKSZN Kft., Festetics György u. 7., Keszthely, Hungary

**Keywords:** Population genetics, Genetic hybridization, Conservation biology

## Abstract

The golden jackal (*Canis aureus*) is a reoccurring species in the centre of the Carpathian basin, in Hungary. In total, 31 golden jackal tissue samples were collected, from 8 white-coated, 2 black-coated and one mottled animal across Hungary. Sequences and fragment length polymorphisms were studied for white colour (MC1R), and for black coat colouration (CBD103). In each white animal, the most widespread mutation causing white fur colour in dogs in homozygous form was detected. Three animals were found to carry the mutation in heterozygous form. The two black golden jackals were heterozygous for the 3 bp deletion in CBD103 that mutation for black coat colouration in dogs, and one of them also carried the mutation causing white fur. None of the white animals showed signs of hybridization, but both the black and the mottled coloured individuals were found to be hybrids based on genetic testing. Kinship was found three times, twice between white animals, and once between a white animal and an agouti animal carrying the mutation of white coat. Our results confirm the findings that golden jackal*–*dog hybrids may occur without human intervention, and the detected mutation causing white fur colour in golden jackals could possibly be due to an early hybridization event.

## Introduction

The golden jackal (*Canis aureus*) is a medium-sized canine leading an opportunistic lifestyle^1^. Its distribution is mainly across Northern Africa, Southern Europe, Near East, India and South Asia, but it is rapidly spreading towards Northern and Western European areas^2^. In Eastern Europe and the Balkan region its presence was continuous during the twentieth century and its numbers were constant^3^, but in Hungary during the 19th and the majority of the twentieth century only stray animals were detected^4–6^. The expansion of the species started from Bulgaria in the 1960s^7^; soon its numbers started to rise in Romania^8^, and there were recordings of golden jackals in Serbia at the end of the 1970s–beginning of 1980s^5^. In Hungary, the first observations of relocation occurred at the beginning of the 1980s^5^, and ever since, its numbers are rising^9^ despite local population management, like trapping and extensive hunting. The golden jackal is generally found in open grasslands, but with urbanization and its opportunistic habitat use, jackals can now be found around human settlements as well^10^.

Genetically, the golden jackal is a close relative of the coyote (*Canis latrans*), the grey wolf (*Canis lupus*), the African wolf (*Canis lupaster*), the African golden wolf (*Canis anthus*) and the dog (*Canis familiaris*)^11^. Phylogenetic and evolutionary studies show that wolves, coyotes and dogs share genomic elements^12^, and there is significant gene flow between different Canid species^13–15^.

In North America, hybridization between the different Canid species has been observed and well documented, and it has been found recently that grey wolves, red wolves, coyotes and Algonquin wolves show little to no genomic differences^16,17^. Even though most wolf species fall under the Endangered Species Act of the USA^18^, there have been several attempts to establish wolf-dog hybrid breeds, such as the Calupoh^19^.

Coyotes are known to interbreed with dogs^20,21^, and genetic analyses of mitochondrial DNA show that dog ancestry is widespread in coyotes in the southeastern United States^14^. In 2010, Kays et al.^22^ found that eastern coyotes interbreed with wolves in the northeastern area of the US. They also found that this hybridization contributed to a larger body size, which resulted in better hunting abilities regarding deer, and the larger coyotes filled a vacant niche in the area left by the eradication of wolves^15,23^. These coyote-wolf hybrids are generally known as coywolves^24^, but whether they should be considered a subspecies is still heavily debated^22^.

Hybridization with dogs has caused several phenotypic changes in wolves, such as black coat colouration in North American grey wolves, which was introduced to the species at least 7500 years ago through introgression with dogs^25^, but there have also been documented cases of hybridization between dogs and coyotes^25^ and wolves in North America^26^ resulting in several morphological changes.

There is no direct evidence of hybridization between grey wolves (*Canis lupus*) and golden jackals in the wild, but there is genetic evidence of admixture between golden jackals and African wolves (*Canis lupaster*) in Israel^27^.

Historical anecdotes^28,29^ can be found about possible jackal–dog hybrids. Erik Ziman described breeding experiments with poodles and jackals^30^, and well-documented evidence for human-made dog–jackal hybrids are the Sulimov dogs^31,32^. Recently in India, camera trap pictures have shown golden jackals showing dog-like pelt colouration in urban areas^33^, which could be due to possible hybridization. One documented instance of hybridization in the wild was confirmed by genetic studies, which was described by Galov et al. in^34^.

Phenotypic changes resulting in pheomelanistic coat due to MC1R mutations are observed in many mammal species^35–37^, but a loss-of-function mutation is also responsible for the white or golden colouration of dogs’ fur colour^38^. The same mutation could also be found in coyotes^39^ in North America.

In dogs, there are two widespread MC1R mutations: the first is the dominant E^m^ (melanistic mask), which is caused by a valine to methionine change at amino acid 264, resulting in a melanistic mask on the face, dark underside and legs^40^. The other is a “recessive yellow” allele “e”, which changes an arginine to a premature stop codon at amino acid position 306, and is considered recessive^38^, therefore can remain hidden for generations without selection^41^. Breed-specific mutations exist in sighthounds^42^, English Cocker Spaniels, huskies and Australian cattle dogs^43^, but they have not been found in wild canids yet.

Black or melanistic coat colour in dogs is caused by a 3-basepair in-frame deletion (ΔG23) in the canine beta-defensine 103 gene (CBD103)^44,45^. This ΔG23 has been found in North American wolves^25^, Italian wolves^46^, coyotes^47^, and in golden jackal-dog hybrids described by Galov et al.^34^. Melanism is considered a dominant trait, therefore one copy of the mutation is enough to create a phenotypic change with a properly working melanocortin-1 receptor.

Merle coat colouration in dogs is caused by the insertion of a short interspersed element (SINE) into the SILV gene and results in diluted areas of eumelanistic fur, showing a mottled phenotype in heterozygous form^48,49^, whereas in homozygous form it associates with ocular abnormalities and deafness^50^. Clark et al. reported that several dogs with non-merle phenotype were heterozygous for a smaller insertion, and Langevin et al. found that length differences in the poly-A tail of the SINE insertion in the SILV gene results in differences in the merle phenotypes of dogs that can be categorized into six groups based on the poly-A tail length (Mc, Mc +, Ma, Ma +, M, Mh)^51^.

Recently there have been several documented and legally shot white-coloured golden jackals in the southern areas of Hungary, as well as black and mottled animals. Our aim in this study was to determine the mutations behind their fur colour, and to detect whether the change in their phenotype was due to possible hybridization with dogs, using microsatellite markers and mitochondrial sequencing.

## Results

### MC1R results

As a reference sequence we used the 954 bp long MC1R sequence of ROS_Cfam_1.0 [Accession number: NC_051809.1:c63923224-63922271].

Four SNPs were present in our samples, c.475C>T, c.476C>A, c.790A>G and c.916C>T; the latter of these is the stop codon mutation for white coat colouration in dogs.

**Figure 1:**
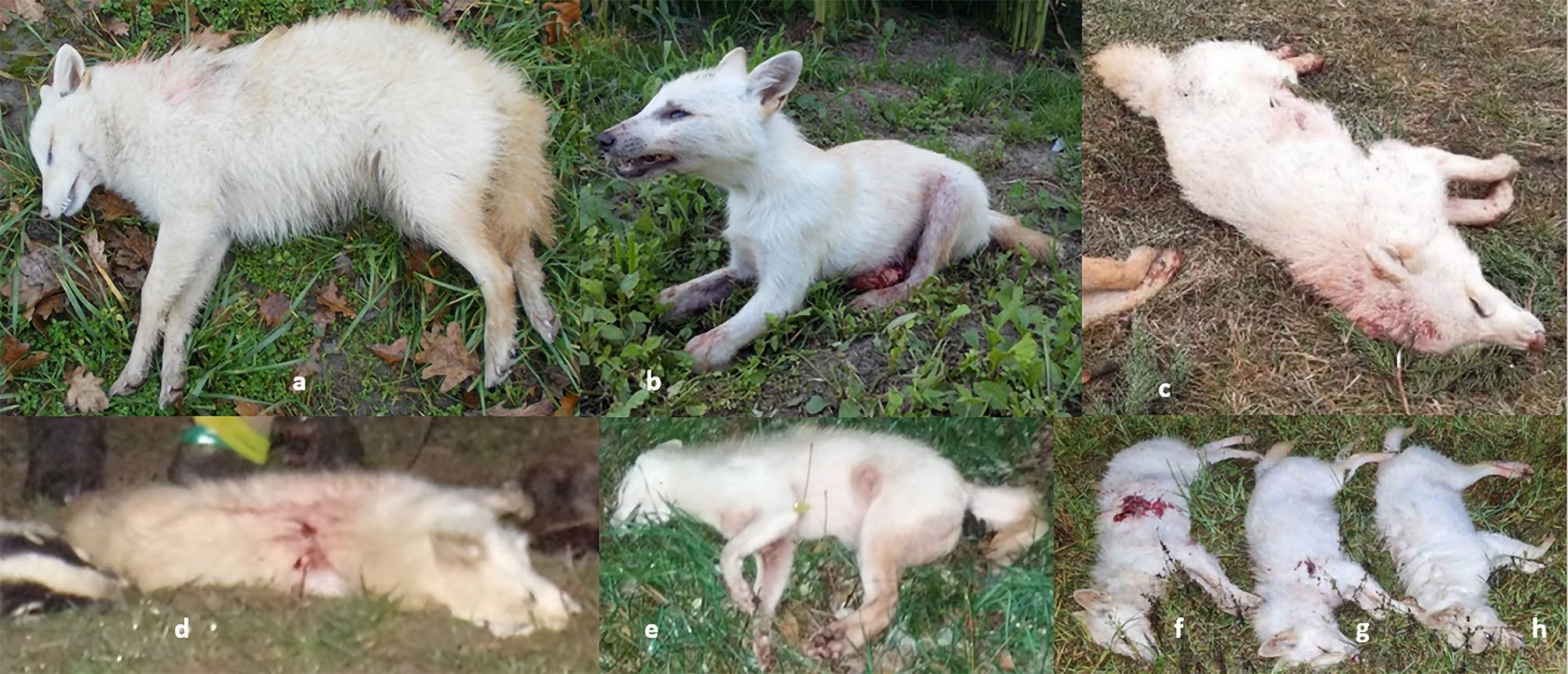
Photographs of the white animals, 4744CaW (**a**), 4798CaW (**b**), 6392CaW (**c**), 6350CaW (**d**), 6343CaW (**e**), 6647CaW (**f**), 6648CaW (**g**), 6651CaW (**h**).

At c.475, where most agouti-coloured golden jackals are homozygous for the T allele, which results in a proline to serine amino acid change, one animal showed heterozygosity. At c.476, white-coloured jackals are all homozygous for the A allele, resulting in a proline to glutamine amino acid change. We found two instances of heterozygosity of c.790A>G, which is the mutation causing masked phenotype in dogs. At c.916, all white animals (Fig. 1) were homozygous for the T allele, which results in a stop codon terminating the open reading frame of the melanocortin receptor gene. We also found three animals heterozygous for the c.916C>T mutation.

Analysing the other previously described SNPs in the MC1R sequences^38^, at Ser90Gly (c.268) all of our samples were homozygous for the A allele, at Ala105Thr (c.313) they were all homozygous for the G allele, and at Arg301Cys (c.901) all samples were homozygous for the C allele (Supplementary Table 1).

### CBD103 results

**Figure 2:**
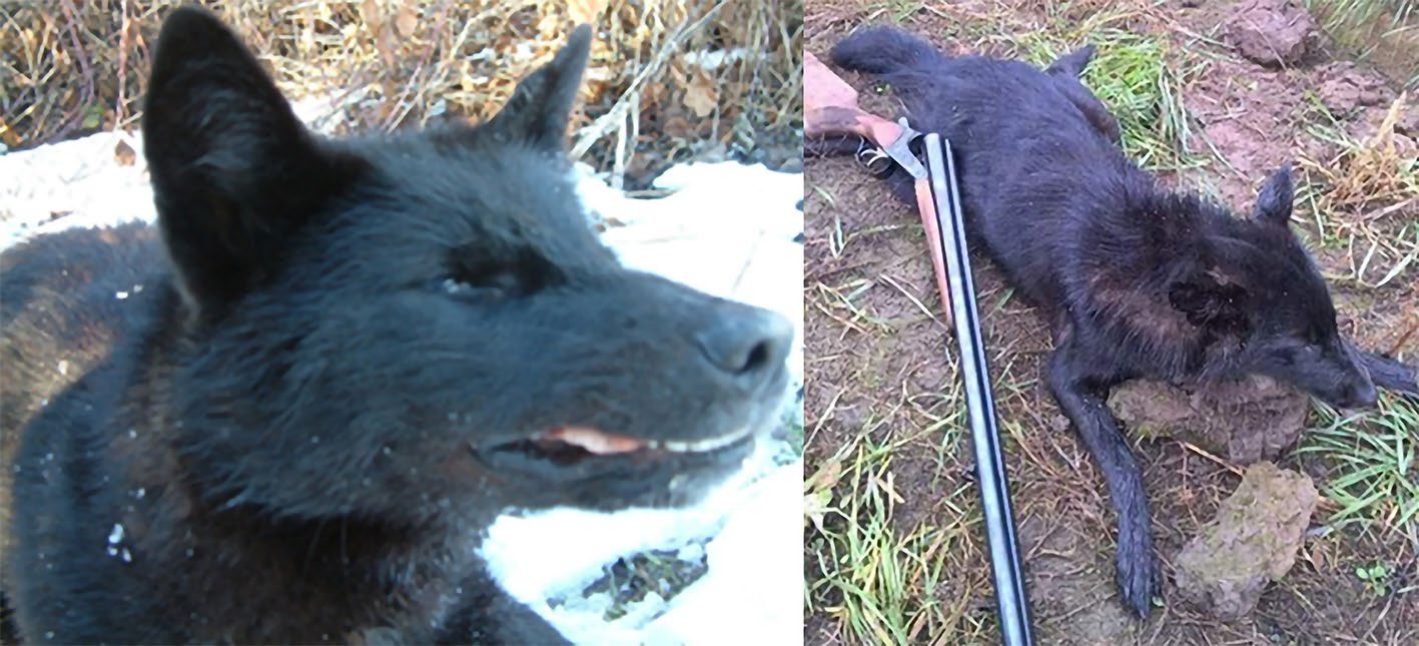
The black animals (5920HybB, 5928HybB) that are heterozygous for the CBD103 deletion.

Not only black, but also white and mottled jackals were genotyped for the ΔG23 mutation of the CBD103 gene, since the 916C>T MC1R mutation is epistatic in homozygous form to this mutation. Using fragment analysis, we found that the black animals (Fig. 2) were heterozygous for the 3-basepair deletion, and all other animals were homozygous for the wild-type allele (Supplementary Table 1).

### SILV results

**Figure 3:**
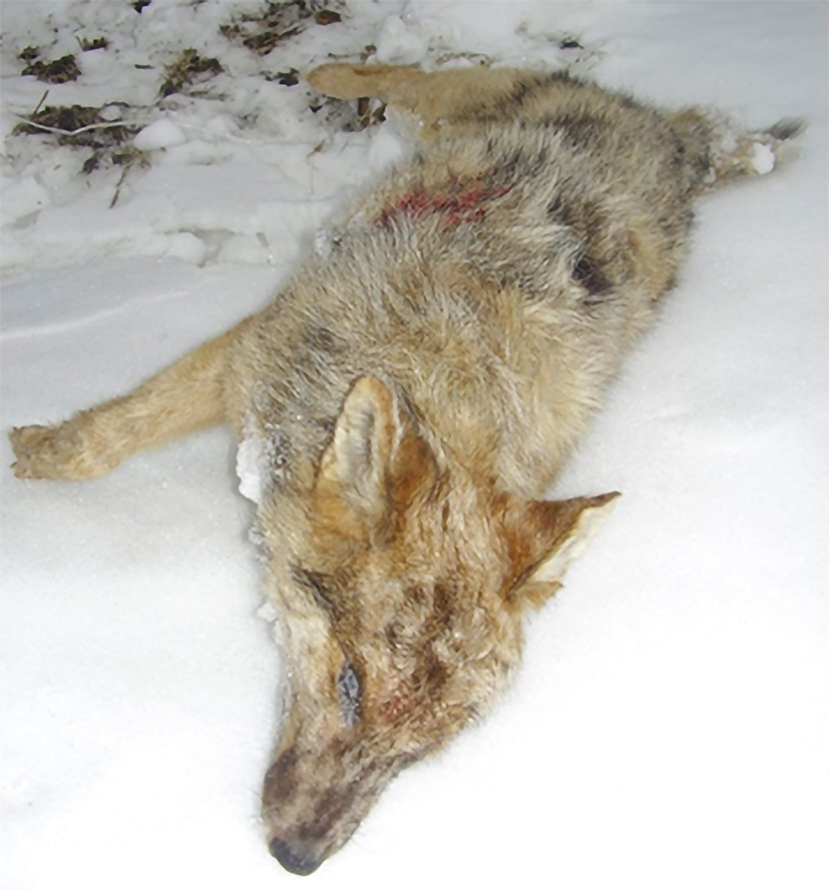
The animal (5930HybM) showing merle phenotype and carrying a SILV SINE insertion.

In the mottled animal’s case (Fig. 3), the phenotype was highly resemblant to agouti or dark sable merle dogs’ look that is caused by a SINE insertion in the SILV gene. Using fragment analysis, we found a peak at 434 basepairs with a wild-type allele of 171 basepairs, which means the animal has a 263-basepair SINE insertion, which categorizes it as Ma +, atypical merle animal.

### Partial mitochondrial DNA sequencing

We analysed the mitochondrial D-loop sequence of the 31 animals acquired for this research using BLAST. In 30 cases the results showed golden jackal origins; sample 5928HybB, however, was identified as a dog (GenBank Accession number: OR047611), which indicates possible dog maternity.

Haplotype analysis revealed that out of the 31 animals in this study, 30 belong to the same haplogroup, with the exception of the animal with dog mitochondria. (Supplementary Table 1). This result confirms the previous findings that golden jackals show a high degree of genetic uniformity^52^.

### Microsatellite analysis, structure and kinship results

**Figure 4:**
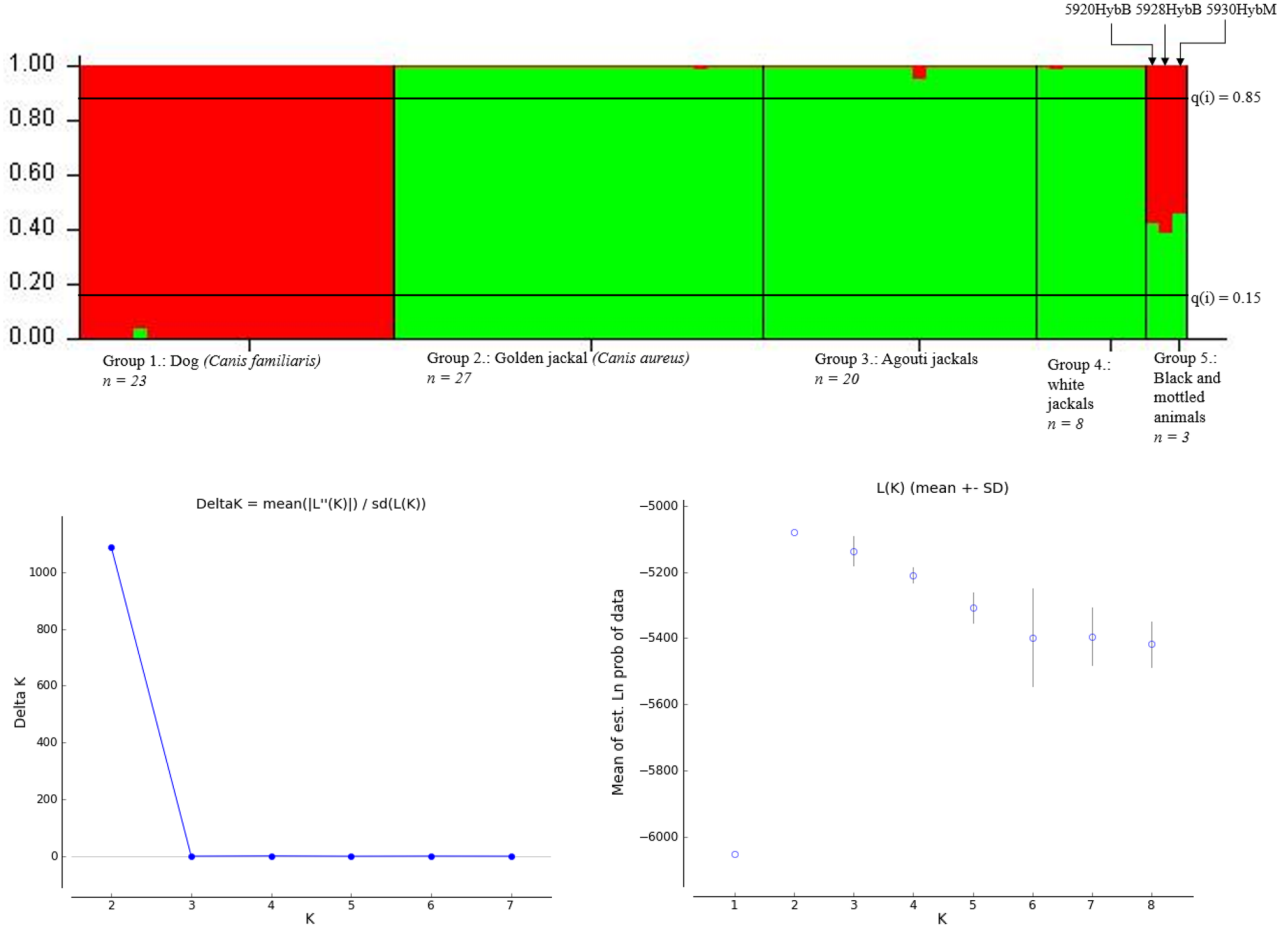
Result of the STRUCTURE analyses at K = 2. Group 1 = reference dogs (n = 23), Group 2 = reference jackals (n = 27), Group 3 = agouti jackals (n = 20), Group 4 = white jackals (n = 8), Group 5 = Black and mottled animals (n = 3). The Delta K value shows the most likely number of clusters, which is 2 in this case. The colour red indicates clustering with the dog group; green is the colour of the golden jackal cluster. In group 5, the individuals show approximately 50% clustering to both groups, so they cannot be clustered into either.

All loci were polymorphic, with the mean number of alleles reaching 5.52, with average expected- and observed heterozygosity being 0.682 and 0.660, respectively (Supplementary Tables 2, 3, 4), all loci were therefore included in subsequent multi-locus analyses. Using 20 polymorphic microsatellite markers, we determined the genetic distances of our samples from dog and golden jackal samples. Structure results showed possible F1 hybridization in three cases (Fig. 4).

At K = 2, the reference populations separated completely, and all animals in the agouti and white group belonged to the jackal population. The two black and the mottled jackals could not be assigned to the reference groups with an assignment probability of 0.15 and 0.85.

**Figure 5:**
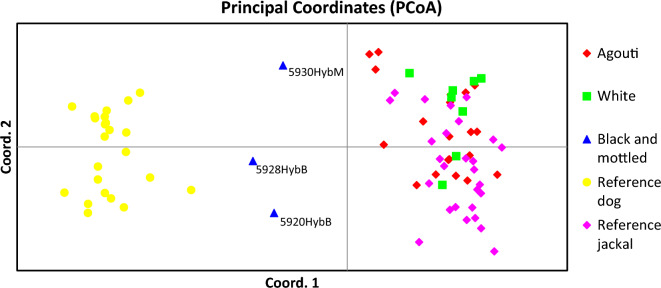
Principal coordinates analysis of the animals in this study. Yellow = reference dogs, pink = reference jackals, red = agouti group, green = white group, blue = black and mottled animals. The white animals are all grouped together with the reference and agouti groups, whereas the coloured animals fall between the reference dog and jackal groups.

Principal Coordinates Analyses show that the white animals are in the same genetic cluster as the agouti-coloured jackals, whereas the black and mottled animals fall between the dogs and golden jackals (Fig. 5).

**Figure 6:**
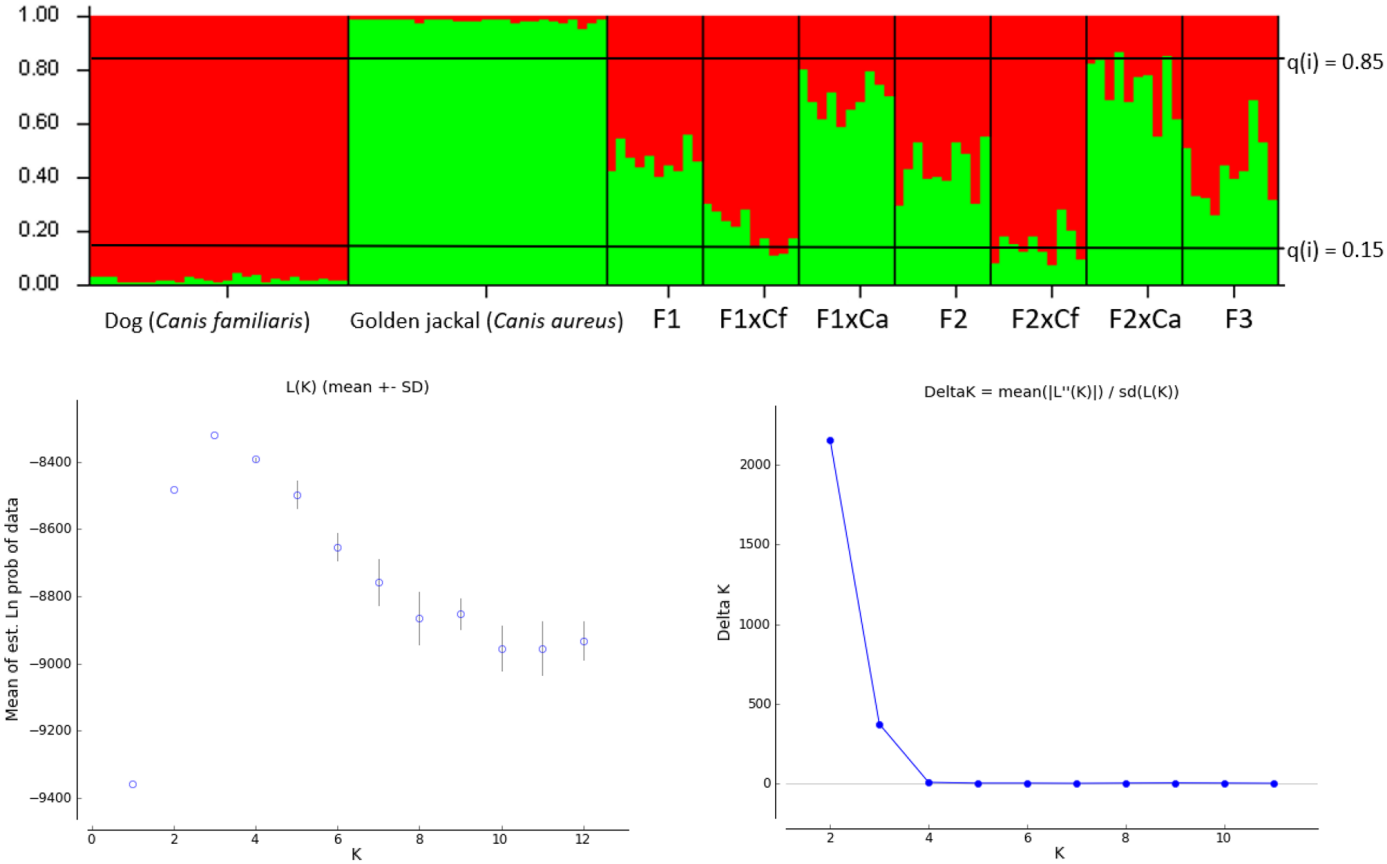
Clustering analysis of the artificial genotypes created by Hybridlab. Group 1: Parental dog (n = 27), Group 2: Parental golden jackal (n = 27), Group 3: F1 (CfxCa) (n = 10), Group 4: F1xDog backcross (n = 10), Group 5: F1xJackal backcross (n = 10), Group 6: F2 (F1xF1) (n = 10), Group 7: F2xDog backcross (n = 10), Group 8: F2xJackal backcross (n = 10), Group 9: F3 (F2xF2) (n = 10). Delta K value shows the most likely number of clusters, which is 2 in this case.

Using STRUCTURE for clustering analysis, we found that none of the artificial genotypes in the F1, F2, and F3 groups could be clustered into either parental cluster. Supplementary Table 5 and Fig. 6 show the results of the artificial genotypes clustering analyses.

We performed kinship analysis with monogamous settings, to determine whether there is a relationship between the white, white carrying, and hybrid animals (n = 13). We found three family clusters between the white and white carrying animals with one likely (Probability > 0.75, 6647CaW/6648CaW/6651CaW) sibling group and two possible sibling groups (Probability > 0.5, 4744CaW/6343CaW and 4802CaA/6392CaW) (Supplementary Table 6).

One cluster (6647CaW/6648CaW/6651CaW) showed close sibling or parent–offspring relationship, which was confirmed by the place and time of the catch—all animals were the same age and killed in the same den at the same time.

## Discussion

With the recent resettlement of golden jackals in the Hungarian lowland, where intensive agricultural involvement and stray or free-ranging dogs are present in areas with low population density, jackals have many opportunities for admixture with local dogs. Since hybridization has not been studied in this area, our research highlights the possibility that there might have been possible earlier hybridization events between dogs and golden jackals.

Analysing the MC1R gene of the animals in this study, we found that the white animals (n = 8) are all homozygous for the R306Ter (c.916C>T) mutation that results in an early stop codon, leading to an entirely pheomelanistic, white colouration, but they are also homozygous for the c.476C>A, a proline to glutamine change. Compared to MC1R sequences downloaded from the NCBI database, we found that the SNP has been described by Wang et al.^53^, and it is present in the Kunming Dog population, as well as in dog breeds from FCI Group 7., Ancient breeds: Alaskan Malamutes, Siberian Huskies, Shar peis, Chowchows, Akitas, Eurasiers, Tibetan mastiffs, it was also found in salukis, beagles and chihuahuas^54^, but it could not be found in other publicly available dog, wolf and coyote MC1R sequences. Since mainly ancient type dogs carry the same mutations as our white samples, it is possible that the “e” allele entered the gene pool through an earlier hybridization event with an ancient type dog.

We also found an additional SNP in the MC1R sequences: at c.475 there is a C>T, proline to serine amino acid change, which appeared only in agouti jackals, and was not found it in the dog, wolf, and coyote MC1R sequences available in the NCBI database.

Galov et al. in^34^ found several wild golden jackal-dog hybrids and confirmed their hybrid status through genetic evidence, supporting the assumption that dogs and golden jackals are capable of interbreeding in areas, where their territories overlap. Stronen et al. proved^16^ that human agricultural intervention can induce hybridization in otherwise sympatric species. Our findings also confirm that hybridization naturally occurs between jackals and dogs, and phenotypic changes can be expected, the most spectacular being the black coat colouration caused by a deleterious mutation in CBD103 originating from dogs, that was found by both Galov et al. and ourselves.

Since the hybrid animals in our study are not relatives, we can here report three different hybridization events, which raises the question how often it happens, and what happened to the littermates of the animals, whether they were unreportedly shot or they are still in the breeding population. The case of the mottled, merle animal sheds light on the dangers of hybridization with dogs—longer SILV SINE insertions in homozygous form can cause developmental issues, like hearing loss and micropthalmia^55^, and since dogs carry at least 152 possible genetic health issues^56^, most of them recessive, developing symptoms after reaching sexual maturity, they are capable of hiding in the gene pool for generations, leading to possible regression of health in isolated populations.

Barash et al.^27^ found evidence for possible hybridization of golden jackals with dogs in Israel that confirms our findings of naturally occurring dog-jackal hybrids, and found introgression with African wolves (*Canis lupaster*), which shows that golden jackals are prone to interbreeding with other Canid species, where their habitats overlap. In Europe, besides free roaming dogs, the only other Canid species golden jackals might share a habitat with are grey wolves (*Canis lupus*), but wolves possibly have a key role in the regulation of the golden jackal population^3^, and we have found no evidence of hybridization between the two species.

Coyotes’ and wolves’ hybridization with dogs has already raised questions regarding the behaviour of the introgressed populations—they become bolder and possibly more dependent on human resources^57^; however, the effects of hybridization on the opportunistic lifestyle of golden jackals is still unknown and needs more investigation.

In conclusion, we identified the mutation responsible for white coat in golden jackals, which is the same as in dogs. This allele was introduced to the species possibly through an earlier hybridization event with an ancient type dog. We also identified three dog-golden jackal hybrid animals, two of them are heterozygous for the CBD103 ΔG23 mutation causing black coat, and one of them is heterozygous for the SILV SINE insertion causing merle pattern in dogs. Our findings confirm that golden jackals interbreed with dogs and hybridization with dogs results in phenotypic change, which in recessive form can stay hidden for generations. A recessive allele for a hereditary disease entering the population from the dogs’ side could pose a danger for isolated jackal populations.

## Methods

### Sampling and genomic DNA extraction

**Figure 7:**
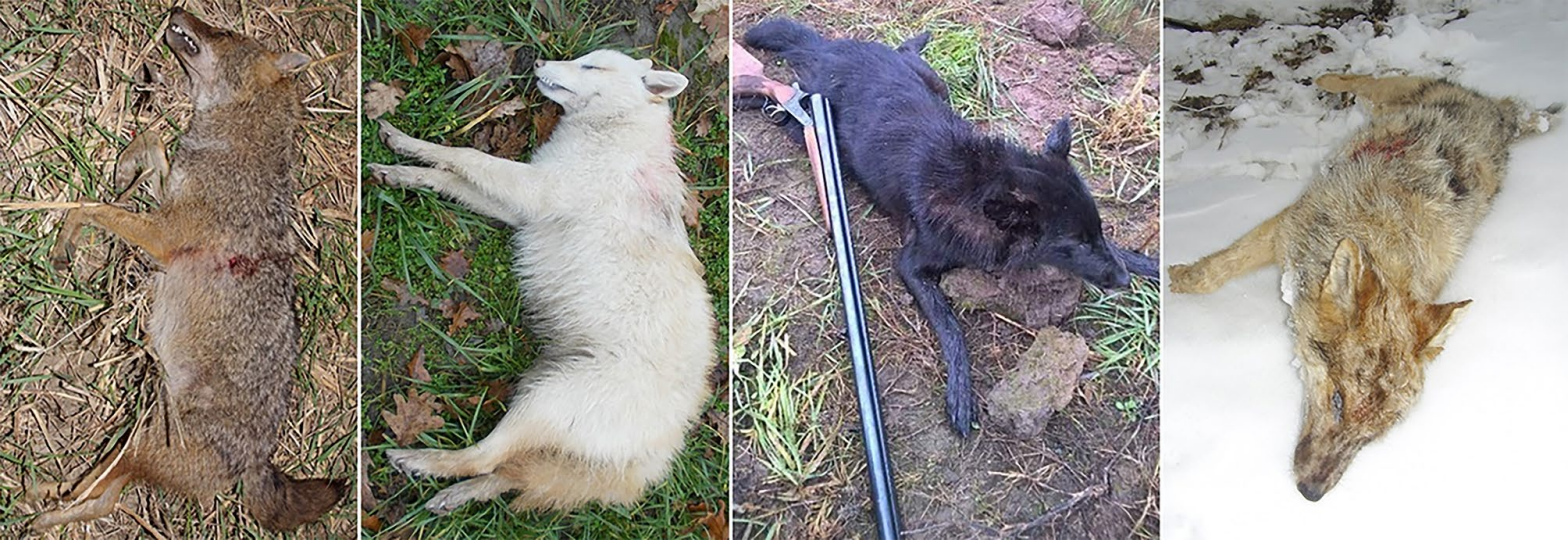
Examples of the coat colourations of the jackals in this study. From left to right: Agouti (sample 6339CaA), white (sample 4744CaW), black (sample 5920HybB) and mottled (sample 5930HybM).

**Figure 8:**
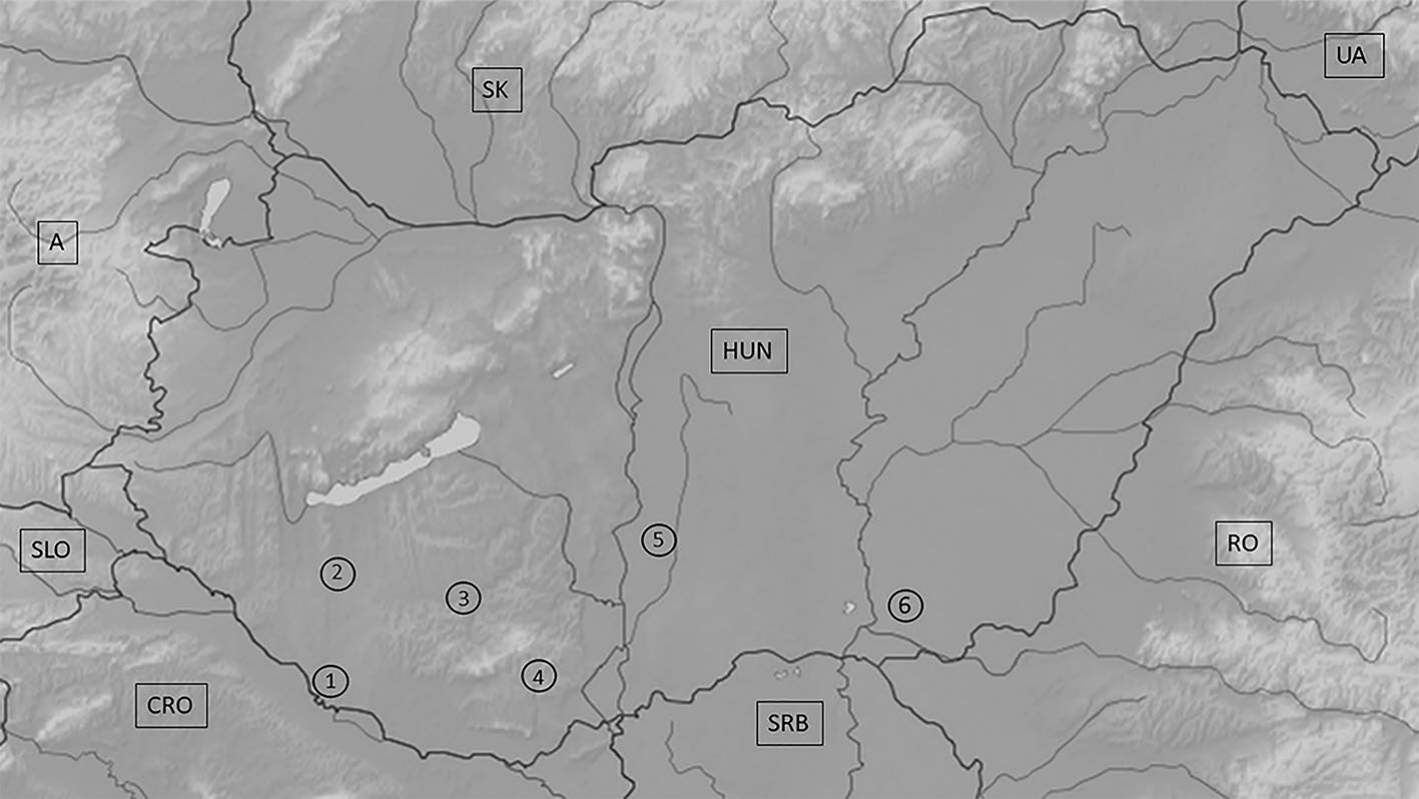
Sampling locations: 1—Inner Somogy Hunting Region (n = 19), 2—South Balaton Hunting Region (n = 1), 3—Zselic—Mid-Somogy Hunting Region (n = 2), 4—Mecsek Hunting region (n = 2), 5—Illancs-Bugac Hunting Region (n = 2), 6—Maros-Csongrád Hunting Region (n = 2). *A* Austria, *SLO* Slovenia, *CRO* Croatia, *SRB* Serbia, *RO* Romania, *UA* Ukraine, *SK* Slovakia, *HUN* Hungary.

**Figure 9:**
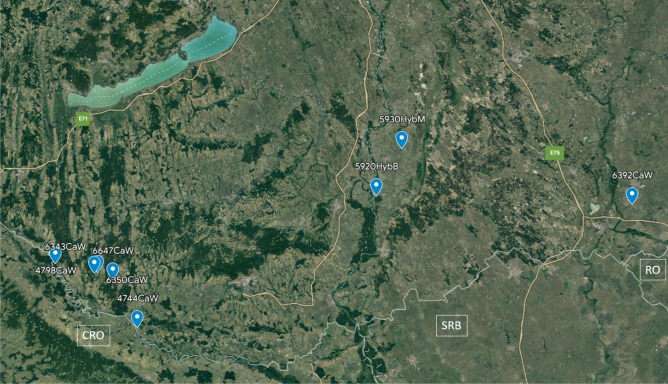
Sample collection sites of the coloured animals. *CRO* Croatia, *SRB* Serbia, *RO* Romania.

Agouti (n = 20), white (n = 8), black (n = 2) and mottled (n = 1) golden jackal (Fig. 7) muscle and liver tissue samples were collected from legally hunted, free-ranging animals between 2014 and 2021 at hunting sites localized in the southern regions of Hungary (Somogy county, Baranya county, Csongrád-Csanád county) (Figs. 8, 9).

As reference samples for studying hybridization levels, hair with follicles (n = 27) from legally shot, free-ranging golden jackals from observed populations, and samples from mixed breed and pedigree dogs (n = 23) were collected and stored at − 20 °C until further laboratory procedure.

Genomic DNA isolation was performed using the MagCore HF16 Automated Nucleic Extractor® with the MagCore Genomic DNA Tissue Kit® (RBC Bioscience Corp., Taiwan), according to the manufacturer’s protocol; the genomic DNA samples were stored at − 20 °C.

### Genetic analysis of colour

MC1R sequencing was performed with D and E primers described by Newton et al.^38^ (primer D: 5′-GGTCATTGCTGAGCTGACAC-3′, primer E: 5′-GAGATGCTGTCCAGTAGTCCC-3′). The volume of the PCR mixture was 25 µl, using Multiplex MasterMix (Qiagen Gmbh, Germany); 0.4 µl of each primer diluted to 10 µM, and 120 ng of DNA diluted to 15 ng/µl. PCR was conducted in a GeneExplorer instrument (Hangzhou Bioer Technology Co., China). The PCR profile was the following: 95 °C for 15 min, 40 cycles of 94 °C for 30 s, 64 °C for 60 s, 72 °C for 2 min, followed by a final extension step of 72 °C for 5 min. The 1347-bp-long PCR products were purified using NucleoSpin® Gel and PCR Clean-up Kit (Macherey–Nagel Gmbh, Germany); Sanger-sequencing was executed using BigDye Terminator 3.1 Cycle Sequencing Kit and 3500xl Genetic Analyzer instrument (Applied Biosystems, USA). MC1R sequences were aligned using SeqMan Pro (DNAStar, Inc), and the alignments were checked manually. We used the MC1R sequence of ROS_Cfam_1.0 (NC_051809.1:c63923224-63922271, Labrador retriever) as a reference.

The MC1R sequences of the samples in this study are available under the Genbank accession numbers OR074050–OR074080.

Analysis of CBD103 was done using primers described by Leonard et al.^44^ (Forward: 5′-(6FAM) TCGGACCAAAGCCTTCCTAAAG-3′, Reverse: 5′-AACGCTCCCATCCCATTTCTGG-3′). The reaction mixture volume was 20 µl, it contained 4 µl of DNA diluted to the concentration of 15 ng/µl, 10 µl of Phire Animal Tissue PCR Buffer, 0.4 µl of Phire HS II DNA polymerase enzyme, 0.5 µl of each primer diluted to 10 µM and 6.6 µl nuclease-free water. PCR was conducted in a GeneExplorer instrument (Hangzhou Bioer Technology Co., China). The PCR profile was the following: 98 °C for 30 s, 35 cycles of 98 °C for 10 s, 55 °C for 10 s, 72 °C for 20 s, and final extension at 72 °C for 5 min. We expected two fragment lengths, the wild-type allele at 228 basepairs and the G23 mutation at 225 basepairs. The 3-basepair deletion in CBD103 was detected by fragment length analysis using Geneious Prime® (Biomatters Ltd).

Amplification of the SILV SINE insertion was done using the method and primers described by Langevin et al.^51^. We used Qiagen Multiplex MasterMix (Qiagen Gmbh, Germany) according to the producer’s instructions. The length of the SINE insertion was determined using Geneious Prime® (Biomatters Ltd).

### Hybridization analyses

To detect possible hybridization with dogs and conclude kinship, we performed microsatellite and mitochondrial sequence analysis on the samples to detect the direction of hybridization.

Amplification of the hypervariable mitochondrial control region (D-loop) was performed with primers described by Fabbri et al.^58^ (5′-TCCCTGACACCCCTACATTC-3′, 5′-CGTTGCGGTCATAGGTGAG-3′) resulting in a 519-basepair amplicon. The reaction mixture volume was 20 µl, it contained 3 µl of DNA diluted to the concentration of 15 ng/µl, 10 µl of Phire Animal Tissue PCR Buffer, 0.4 µl of Phire HS II DNA polymerase enzyme, 0.6 µl of each primer diluted to 10 µM and 5.4 µl nuclease-free water. PCR was conducted in a GeneExplorer instrument (Hangzhou Bioer Technology Co., China). The PCR profile was the following: 98 °C for 30 s, 35 cycles of 98 °C for 20 s, 62.5 °C for 30 s, 72 °C for 70 s, and final extension at 72 °C for 5 min. PCR products were purified using NucleoSpin® Gel and PCR Clean-up kit (Macherey–Nagel Gmbh, Germany); Sanger-sequencing was executed using BigDye Terminator 3.1 Cycle Sequencing Kit and 3500xl Genetic Analyzer instrument (Applied Biosystems, USA).

For microsatellite analysis we amplified 20 loci in 4 multiplexed reactions using Qiagen Multiplex PCR Kit (Qiagen Gmbh, Germany): AHTH137, AHTH171, AHTH260^59^, FH2538, c2001, c2096, c2054, PEZ8, PEZ5, PEZ19, PEZ12, PEZ11^60^, FH3377, FH2088, FH3313, PEZ02, FH2010, FH2107, FH2004, FH2309^61^.

The sex of the animals was identified by hunters and confirmed with the amelogenin gene^62^.

The reaction mixture volume was 25 µl, containing Multiplex MasterMix (Qiagen Gmbh, Germany), 120 ng of DNA, optimalized amounts of primers at 10 µM concentration, and nuclease-free water. PCR was conducted in a GeneExplorer instrument (Hangzhou Bioer Technology Co., China). The PCR profile for all multiplexes was the following: 96 °C for 15 min; 40 cycles of 95 °C for 30 s, 58 °C for 60 s, 72 °C for 60 s, and final extension at 60 °C for 30 min. Fragment capillary analysis was conducted on 3500xl Genetic Analyzer instruments with LIZ500 size standard (Applied Biosystems, USA).

The lengths of microsatellite fragments were described using Geneious Prime® (Biomatters Ltd) with Two Surrounding Peaks sizing method.

Mitochondrial sequences were aligned using SeqMan Pro (DNAStar, Inc), and the alignments were checked manually. For species identification, the sequences were loaded into the Basic Local Alignment Tool (BLAST®)^63^. Haplotype analyses were carried out using MEGA11^64^. The mitochondrial D-loop sequences of the samples in this study are available under the Genbank accession numbers OR047611–OR047641.

### Statistical analysis

For genetic clustering we used STRUCTURE ver. 2.3.4.^65^; for analysis of STRUCTURE results, we used STRUCTURE Harvester v06.94^66^.

The Bayesian clustering method in STRUCTURE was used to ascertain the number of genetic clusters to define possible admixture of golden jackals and dogs. The Bayesian clustering method and the Markov Chain Monte Carlo (MCMC) simulation were run assuming no prior information and using an admixture and independent allele frequency models. The simulation was run with 7 independent runs for each K value ranging from 1 to 9, with a burn-in period of 250,000 iterations and 750,000 replications. The number of genetic clusters (K) was calculated with the second-order rate of change in log-likelihood values (ΔK) in STRUCTURE Harvester v06.94^66^.

Genetic distance analysis and Principal Coordinates Analysis were performed using GenAlEx 6.5^67^.

To determine whether our microsatellite set is capable of distinguishing F1 or F2 hybrids, we created artificial genotypes using Hybridlab^68^ in the following iterations: parent clusters (dog and golden jackal), F1 (dog × jackal), dog and F1 hybrids, jackal and F1 hybrids, F2 (F1 × F1), dog and F2 hybrids, jackal and F2 hybrids and F3 (F2 × F2) hybrids. We created artificial parent populations with 27 genotypes each, and artificial offsprings with 10 genotypes each.

Kinship analysis was based on fragment length and was done using Colony v2.0.6.6^69^ and Cervus 3.0.7^70^.

### Ethics statement

This study was out of the scope of the 2010/63/EU Directive, and was carried out with the knowledge and permission of the Institutional Animal Welfare Committee of Hungarian University of Agricultural and Life Sciences Szent István Campus (MATE-SZIC/1068-1/2023).

### Supplementary Information

Supplementary Legends.

Supplementary Table 1.

Supplementary Table 2.

Supplementary Table 3.

Supplementary Table 4.

Supplementary Table 5.

Supplementary Table 6.

## Data Availability

The genetic sequences of the samples have been deposited in the public sequence repository NCBI GenBank, the mitochondrial control region (D-loop) sequences are available under accession numbers OR047611–OR047641, the MC1R sequences are available under the accession numbers OR074050–OR074080.
