# Supplementary material for: White and other fur colourations and hybridization in golden jackals (Canis aureus) in the Carpathian basin
Source: Sci Rep. 2023 Dec 11;13:21969. doi: 10.1038/s41598-023-49265-0 (PMC10713657; doi:10.1038/s41598-023-49265-0)
Supplement: Supplementary file 1 — Supplementary Legends. [file 41598_2023_49265_MOESM1_ESM.docx]

Supplementary table 1: Summary of the details, colour genetic and clustering analysis results of the samples in this study

Supplementary table 2: Genetic variability at 20 autosomal microsatellites in domestic dogs and golden jackals sampled in Hungary

Supplementary table 3: Allele Counts and Frequencies by populations

Supplementary table 4: Allelic patterns by populations

Supplementary table 5: Percentage of unclassified genotypes created by Hybridlab. None of the genotypes in F1, F2 or F3 could be classified into either parental group (0.15<(q(i)<0.85).

Supplementary table 6: Percentage of family cluster probability of related samples. Relationship between samples is possible if the probability is higher than 50%, and likely if the probability is higher than 75%.
